# Inflammatory and neurotoxic risk of atorvastatin in diabetic peripheral neuropathy: TNF-centered evidence integrating network toxicology, scRNA-Seq, and cell validation

**DOI:** 10.3389/fchem.2026.1739085

**Published:** 2026-02-18

**Authors:** Hanbing Yang, Jing Chen, Peilin Zhu, Meng Yuan, Siyu Mao, Yujun He, Xin Wang, Jingni Wang, Xingchun Wang, Xingxia Wang

**Affiliations:** 1 Renshou County People’s Hospital, Meishan, Sichuan, China; 2 Department of Neurology, The Affiliated Hospital, Southwest Medical University, Luzhou, Sichuan, China; 3 Department of Clinical Medicine, School of Clinical Medicine, Southwest Medical University, Luzhou, Sichuan, China; 4 The Affiliated Stomatological Hospital, Southwest Medical University, Luzhou, Sichuan, China

**Keywords:** atorvastatin, DPN, molecular docking, network toxicology, single-cell RNA sequencing, TNFα

## Abstract

**Objective:**

To clarify atorvastatin’s role in diabetic peripheral neuropathy (DPN) amid its controversial neuroprotective and neurotoxic effects.

**Methods:**

Integrated network toxicology, single-cell RNA sequencing (scRNA-seq), molecular docking, molecular dynamics simulations, and *in vitro* assays (CCK-8, ELISA) on high-glucose-induced RSC 96 Schwann cells.

**Results:**

Network toxicology identified TNF, CTNNB1, CASP3 as core targets (TNF as key hub), enriched in DPN-related pathways (oxidative stress, inflammation). scRNA-seq suggested that these targets are expressed in sensory neuron populations. Molecular docking and molecular dynamics simulations suggested that atorvastatin can interact with the selected targets, with relatively favorable predicted affinity for TNFα. *In vitro*, atorvastatin reduced cell viability in a time- and dose-dependent manner and was associated with increased TNFα levels under high-glucose conditions.

**Conclusion:**

Our findings are consistent with a potential involvement of TNF/TNFα-associated inflammatory responses in atorvastatin-related cellular injury under the tested *in vitro* conditions. Further TNFα blocking/knockdown experiments will be needed to determine causality.

## 1 Introduction

Diabetes has emerged as a global health crisis, with its prevalence reaching unprecedented levels and imposing a heavy burden on healthcare systems worldwide. Among the complications of diabetes, diabetic peripheral neuropathy (DPN) is one of the most common and disabling microvascular complications, affecting up to 50% of diabetic patients (Dilshad and Ephrem, 2023), while its estimated prevalence among U.S. adults with diabetes is 28% (Hicks and Selvin, 2019). This progressive neurological disorder is characterized by distal symmetric polyneuropathy and manifests through symptoms such as numbness, burning pain, and motor dysfunction (Sanaye and Kavishwar, 2023). These symptoms not only severely impair patients’ quality of life but also increase the risk of foot ulcers, which often progress to amputations, further worsening disability and even being life-threatening (Volmer-Thole and Lobmann, 2016).

The damage of peripheral nerve by metabolics dysfunction in the late stages leads to DPN to occur (Zhu et al., 2024). Hyperglycemia, which is typical for diabetes mellitus, causes a number of harmful biological processes and excites the excessive production of reactive oxygen species (ROS). Glucose-induced oxidative stress hampers cell’s antioxidant mechanisms and damages lipids, proteins and DNA in neurons (Lee et al., 2020). Constant increase in blood glucose facilitates the accumulation of advanced glycation end products (AGEs), produced by non-enzymatic reactions between sugar and protein. Activation of the pro-inflamatory pathways and neuroinflammation by AGEs ligated to the receptor represents one of the main mechanisms contributing to the nerve damage. In this inflammatory context, tumor necrosis factor-α (TNFα) has been implicated in the development of DPN. A meta-analysis reported elevated circulating TNFα levels in patients with DPN compared with diabetic individuals without neuropathy, supporting an association between TNFα and DPN. Moreover, experimental inactivation/neutralization of TNFα in streptozotocin-induced diabetic mice ameliorated neuropathy-related functional deficits and was accompanied by changes in downstream inflammatory signaling (including NF-κB-related readouts), suggesting that TNFα-associated inflammation may contribute to DPN pathophysiology. Therefore, TNFα represents a plausible mediator linking metabolic stress to inflammation-related peripheral nerve injury, although the causal contribution of TNFα may depend on disease stage and experimental context (Mu et al., 2017; Yamakawa et al., 2011). Mitochondrial dysfunction also has an important role, besides blocking energy production, the latter activates ROS production (Bai and Luo, 2024). They disrupt axonal transport, impair neurovascular endothelial function, and accelerate demyelination (Cardoso and Salles, 2016; Wu et al., 2013).

Atorvastatin, an inhibitor of 3-hydroxy-3-methylglutaryl-coenzyme A (HMG-CoA) reductase, is widely used to lower lipids and cardiovascular risk in hypercholesterolemia (Lee et al., 2020; Meng et al., 2019). Despite there is abundant evidence that atorvastatin is causes neurological adverse reactions, diabetic patients are concerned that prolonged or high-dose use could lead to the exacerbation or progression of peripheral neuropathy (Evangelista et al., 2019). It seems that the atorvastatin neurotoxicity could manifest itself in various aspects. In addition to increasing oxidative stress via ROS overproduction in the neuronal cells, the loss of mitochondrial homeostasis and maintenance of the cellular metabolism are revealed. Inhibition of the cholesterol synthesis in neurons’ membranes and the membranes integrity are involved (Matoba et al., 2019; Xie et al., 2023). These combined factors worsen nerve damage in diabetic patients and can initiate or speed up DPN. A person’s susceptibility to this damage depends on drug interactions, genetic predisposition, and overall health (Meng et al., 2019). Detailed assessment of potential neurotoxicity of atorvastatin is necessary to better understand its benefits when applied to patients with DPN.

Network toxicology is a relatively advanced approach that integrates multi-omics data, protein-protein interaction (PPI) networks, and pathway analysis, providing a very useful framework for addressing such complex situations. By aggregating and analyzing large-scale datasets, it can identify key molecular targets and signal cascades mediating drug effects, which are introduced in references (Lin et al., 2021; Wu et al., 2023). This method has been successfully used to clarify the toxicological mechanisms of environmental pollutants and drugs, such as muscle-related adverse reactions induced by statins. Applying network toxicology to the research of atorvastatin and DPN enables researchers to systematically depict the interactions between drug targets and molecules involved in the pathogenesis of DPN, such as molecules regulating oxidative stress, apoptosis and neurotrophic factor signaling. These molecules support the survival and repair of neurons. In addition, molecular docking can predict the binding affinity of atorvastatin and key targets related to DPN, which can select the most likely biologically relevant interactions that are worthy of experimental verification (Sun et al., 2023). This synergy between computational and experimental methods bridges the gap between *in silico* analysis and *in vivo* significance, and accelerate the discovery of actionable mechanisms.

As a supplementary content to network toxicology and molecular docking methods, single-cell RNA sequencing (scRNA-seq) has become a revolutionary tool. It can study complex biological processes and disease mechanisms with resolution never seen before (Awuah et al., 2023). By capturing the complete transcriptome expression profile at the single-cell level, scRNA-seq has solved the limitations of bulk sequencing and it will average the gene expression in cell populations and mask the heterogeneity within tissues. This technology can describe the gene expression patterns within individual cells, thereby revealing the functional diversity specific to cell types, identifying rare or previously undiscovered cell subpopulations, and depicting the dynamic transcriptional changes related to drug exposure or disease progression. With the continuous development of gene chips and bioinformatics technologies, scRNA-seq data are widely used in disease research to crack complex pathogenic mechanisms. In the context of neurological disorders such as DPN, scRNA-seq offers a particular opportunity to determine how atorvastatin regulates gene expression in different cell types involved in peripheral nerve homeostasis, including neurons, Schwann cells, and vascular endothelial cells, etc. It can also provide insights into the mechanisms by which it functions at the cellular level.

**FIGURE 1:**
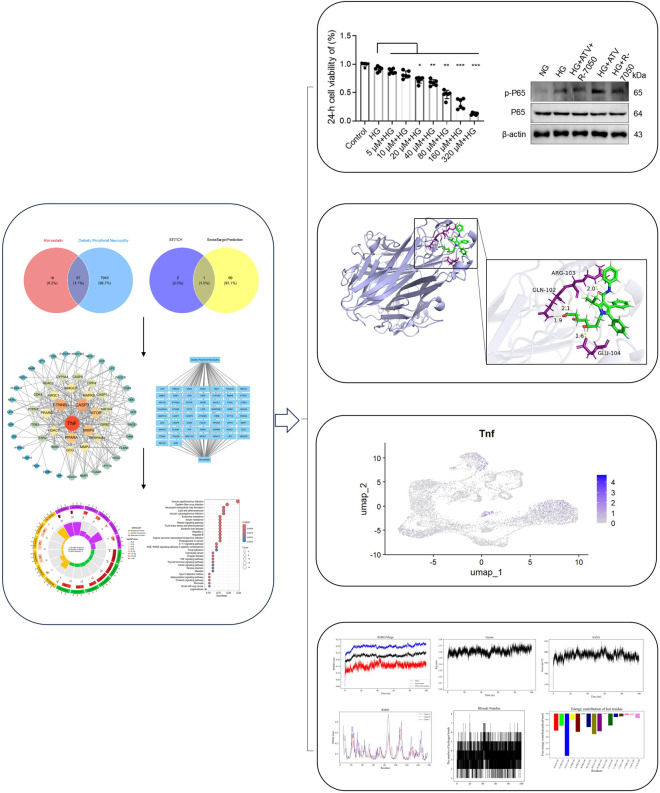
Schematic representation of the methodological framework and analytical pipeline implemented in the present investigation.

This study aims to integrate network toxicology, molecular docking, molecular dynamics simulation, scRNA-seq and other methods to comprehensively explore the possible effects of atorvastatin on DPN (Figure 1). To clarify the potential molecular mechanisms behind the various effects of atorvastatin on peripheral nerves, multiple research strategies have been adopted. This includes conducting systematic network toxicology-based studies on the interaction between atorvastatin and its biological targets, using molecular docking to predict the binding affinity between the drug and the target, verifying the kinetic stability of the conjugate through molecular dynamics simulation methods, and describing the transcriptional responses specific to cell types using single-cell RNA sequencing. The toxicity of atorvastatin on peripheral neuro-related cells was verified in an *in vitro* cell model by the CCK-8 method, and cell experiments were conducted to confirm the mediating role of related molecules in atorvastatin-induced DPN, deepening our understanding of the role of atorvastatin in DPN and provided more precise and personalized treatment strategies for this intractable complication. Clarifying this complex relationship can create conditions for improving the care of millions of diabetic patients and those with neurological sequelae worldwide.

## 2 Materials and methods

### 2.1 Network toxicology

The direct protein targets of atorvastatin were predicted using the STITCH database (http://stitch.embl.de/), which specializes in protein-chemical interactions, and the SwissTargetPrediction database (https://www.swisstargetprediction.ch/), respectively. The intersection of targets from the two databases was obtained to minimize false positives and define reliable drug targets. Genes associated with DPN were retrieved from the GeneCards database (https://www.genecards.org/) and OMIM database (https://omim.org/) using “diabetic peripheral neuropathy” as the search query. Duplicate entries were then removed to generate a comprehensive list of DPN-related genes.

Subsequently, R 4.4.3 was launched, and the “VennDiagram” package was installed and loaded. Separate datasets for atorvastatin targets and DPN-related genes were created, and Venn diagram analysis was performed to identify common targets. These overlapping targets were then uploaded to the STRING database (https://string-db.org/), and used to construct a PPI network, limiting the search to *Homo sapiens* with a confidence score ≥0.4. The resulting PPI data was imported as a tab-separated values file (.tsv) into Cytoscape v3.10.2(https://cytoscape.org/). Two additional nodes, ‘Atorvastatin’ and ‘DPN,’ were manually added and connected to their respective targets within the network to improve visualization.

To further refine the PPI network, the overlapping targets were re-analyzed in STRING database, retaining only interactions with a confidence score ≥0.4. Protein interaction data were then exported as a ‘protein_links.tsv’ file and imported into Cytoscape v3.10.2 for network visualization and analysis. A PPI network was algorithmically generated by defining source nodes, target nodes, and edge attributes.

Network topological parameters were analyzed utilizing the “Network Analyzer” plugin in Cytoscape v3.10.2. The resulting dataset was exported to Excel for calculation of average node degree. Nodes were sorted in descending order based on degree values, and a threshold was applied to identify core targets. The node exhibiting the highest degree was designated as the network’s central hub, with the number of connected edges recorded. An annotated screenshot of the network was captured, highlighting these core targets.

Gene symbols of core targets were converted to Entrez Gene IDs using the “clusterProfiler” and “org.Hs.eg.db” packages in R 4.4.3. These Entrez Gene IDs were uploaded to the DAVID database (https://david.ncifcrf.gov/) and categorized under the “ENTREZ_GENE_ID” identifier type. On the “Functional Annotation” interface, GO categories were selected, and significantly enriched terms were identified using thresholds of *P* < 0.05 and false discovery rate (FDR) < 0.05. Similarly, the “KEGG_PATHWAY” option was utilized to detect significantly enriched pathways under identical statistical criteria.

Results were exported, and bubble plots were generated in R 4.4.3 using the “ggplot2” package, where bubble dimensions corresponded to the number of enriched genes and color intensity reflected FDR values. These visualizations were archived for subsequent analytical procedures.

### 2.2 Single-cell sequencing

Single-cell level bone transcriptomic data from mouse models of T2DM were downloaded from the GEO database (https://www.ncbi.nlm.nih.gov/geo/query/acc.cgi?acc=GSE272612). Notably, GSE272612 is a bone tissue single-cell transcriptomic dataset from mouse T2DM models. In this study, we used this dataset as an exploratory, hypothesis-generating resource to examine cell-type–associated expression patterns of candidate genes. Therefore, the scRNA-seq results should not be interpreted as direct evidence for gene localization in peripheral nerve tissue. First, the Read10X function was used to read 10X data of the group, and the CreateSeuratObject function (with parameters set to min.cells = 3 and min.features = 200, and sample groups used as project names) was applied to construct individual Seurat objects. All Seurat objects were then merged using the merge function (with add.cell.ids to label sample sources), and after integrating data layers via JoinLayers, the Group information was added to the meta.data of the Seurat object using the match function (Franzén et al., 2019). Subsequently, the PercentageFeatureSet function (pattern = “^mt-”) was used to calculate the percentage of mitochondrial genes in cells, which was stored in the percent.mt column of meta.data; low-quality cells were filtered using the subset function with the criterion of nFeature_RNA >200 and nFeature_RNA <4,000. During data preprocessing, normalization was conducted using the NormalizeData function (method: “LogNormalize”, scale.factor = 10,000); highly variable genes were screened via the FindVariableFeatures function (method: “vst”, nfeatures = 2000); and the expression matrix of highly variable genes was standardized and centered using the ScaleData function to prepare for principal component analysis (PCA). In dimensionality reduction and clustering analysis, PCA was performed based on highly variable genes using the RunPCA function, and the PCA results were visualized by the DimPlot function with coloring by Group. The ElbowPlot function was used to analyze PCA inflection points, and 1–15 principal components (PCs) were determined for clustering. The FindNeighbors function (dims = 1:15) was used to construct a cell adjacency matrix, and cell clustering was conducted using the FindClusters function (resolution = 0.5). UMAP dimensionality reduction was performed via the RunUMAP function (dims = 1:15), and the UMAP clustering results were visualized using the DimPlot function (label = TRUE, pt.size = 0.1, legend hidden). For marker gene identification, the FindAllMarkers function (only.pos = TRUE, min.pct = 0.25, logfc.threshold = 0.25, return.thresh = 0.05) was used to screen significant highly variable genes for each cluster. Top 10 marker genes per cluster were selected based on avg_log2FC by grouping by cluster (group_by(cluster)), and these genes were saved as “top.markers.csv” and “pbmc.markers.csv”. In the cell type annotation stage, a cluster-cell type correspondence list was constructed manually based on marker genes and known cell type markers from the PanglaoDB databases (Franzén et al., 2019), where 16 clusters were annotated as neuronal cluster (neuronal-like), Oligodendrocyte, Astrocytes, EC, Pericytes, Smooth muscle c., Macrophages, Fibroblasts, and Neutrophils. Cell types were renamed using the RenameIdents function, and cell type information was added to the celltype column of meta.data via the mutate function. The UMAP annotation results were visualized using the DimPlot function (label = TRUE, pt.size = 0.1, repel = TRUE, legend hidden, title: “Cell Type Annotation Results”). Finally, the FeaturePlot function was used to display the expression distribution of TNF, CTTNB1, and CASP3 genes in single cells, so as to intuitively present the expression characteristics of target genes in different cell types. These plots are presented to illustrate expression patterns within this dataset and are intended for contextual support rather than definitive tissue-specific localization.

### 2.3 Molecular docking

Structural preprocessing encompassed both ligand and receptor preparation. The ligand atorvastatin(SMILES:CC(C)C1 = C(C(=C(N1CC[C@H](C[C@H](CC(=O)O)O)O)C2 = CC = C(C=C2)F)C3 = CC = CC = C3)C(=O)NC4 = CC = CC = C4) was imported into PyMOL 2.2.0, and crystallographic water molecules as well as non-essential atoms were removed. Hydrogen atoms were added and Gasteiger charges were assigned using AutoDockTools 1.5.7. Rotatable bonds were identified and preserved, with torsion degrees of freedom verified using the Torsion Tree tool. The fully prepared ligand was subsequently exported in “.pdbqt” format.

Crystal structures of the receptors CTNNB1 (β1-Catenin, PDB ID: 1TNF), CASP3 (Caspase-3, PDB ID: 2DKO), and TNFα (Tumor Necrosis Factor-α, PDB ID: 3FQN) were downloaded from the RCSB Protein Data Bank (https://www.rcsb.org/). For each receptor (CTNNB1, CASP3, and TNFα), the corresponding crystal structure was imported into PyMOL 2.2.0. Non-protein components, including co-crystallized ligands, counterions, and bulk solvent molecules, were removed; only conserved water molecules for maintaining the active site structure were retained. The purified protein structures underwent further processing in AutoDockTools 1.5.7: hydrogen atoms were added, protonation states adjusted to reflect physiological pH (7.4), and Gasteiger charges calculated. All atomic bonds were fixed prior to exporting the final receptor structures in PDBQT format.

Docking parameters and execution proceeded as follows. Based on crystallographic data and prior functional studies, binding pocket locations for CTNNB1, CASP3, and TNFα were mapped, with their center coordinates defined in AutoDockTools 1.5.7. A grid box was centered on each active site, and its dimensions were adjusted to fully encompass the entire binding region. A grid spacing of 0.375 Å was employed to ensure adequate sampling of ligand conformations while minimizing computational redundancy. The “exhaustiveness” parameter was set to 32 to balance search depth and computational efficiency, with other parameters maintained at their optimized defaults.

Following docking initiation, docking poses with a appropriate binding energy were initially selected. Ligand-receptor interaction modes were visually inspected using PyMOL 2.2.0. The optimal final pose for each receptor was determined based on three criteria: the lowest binding energy, stable interactions with key residues reported to mediate ligand binding, and the absence of significant steric hindrance. The selected conformations were then exported for subsequent analyses.

### 2.4 Molecular dynamics simulations

To investigate the binding interactions between the receptor and ligand, molecular dynamics simulations of the protein-ligand complex were conducted using GROMACS 2020.3 software (Van Der Spoel et al., 2005). The amber99sb-ildn force field and General Amber Force Field (GAFF) were applied to generate the parameters and topological structures for the protein and ligand, respectively. The simulation box was dimensioned such that each atom of the protein maintained a distance greater than 1.0 nm from the box edges. SPC216 water molecules were used to fill the box, with a portion replaced by Na^+^ and Cl^−^ ions to ensure the system’s electrical neutrality. The steepest descent algorithm was used to minimize the entire system, thus eliminating unfavorable atomic contacts and overlaps. For adequate pre-equilibration, 100-picosecond (ps) simulations of the NVT and NPT ensembles were performed at 300 K (K) and 1 bar, respectively. This was followed by a 100-nanosecond (ns) molecular dynamics simulation under periodic boundary conditions. The temperature (300 K) and pressure (1 bar) were controlled using the V-rescale and Parrinello-Rahman methods, with an integration step of two femtoseconds (fs). Long-range electrostatic interactions were computed via the Particle Mesh Ewald (PME) method, with a Fourier spacing of 0.16 nm (nm). All bond lengths were constrained using the LINCS algorithm. Trajectory visualization, analysis, and animation were carried out using VMD 1.9.3 and PyMOL 2.2.0. The binding free energy of the complex was determined using gmx_mmpbsa.

### 2.5 Cell viability assay

Rat Schwann cells (RSC96; iCell, China) were maintained in DMEM containing 10% fetal bovine serum (FBS) and 1% penicillin–streptomycin at 37 °C with 5% CO_2_. To mimic a diabetic condition *in vitro*, cells were cultured in high-glucose medium (HG, 30 mM), while normal glucose (NG, 5.5 mM) was used as the control. Atorvastatin (ATV) was added where indicated at final concentrations of 5, 10, 20, 40, 80, 160, or 320 μM.

For viability measurements, cells were seeded into 96-well plates and allowed to attach for 24 h. The medium was then replaced with NG or HG medium with or without ATV, and cells were incubated for 24 h or 48 h. After treatment, the supernatant was removed and 100 μL serum-free DMEM was added to each well, followed by 10 μL CCK-8 solution (Beyotime Biotechnology). Plates were protected from light and incubated for 1.5 h, and absorbance was read at 450 nm using a microplate reader. Each condition was run in six wells per experiment. Results are presented as mean ± SD from at least three independent experiments.

### 2.6 Enzyme-linked immunosorbent assay

TNFα levels in culture supernatants were measured using a commercial ELISA kit according to the manufacturer’s protocol. Briefly, after the indicated treatments, cell culture media were collected and centrifuged at 12,000 × g for 10 min at 4 °C to remove cells and debris. The clarified supernatants were then used for ELISA.

A standard curve was generated using serial dilutions of the provided TNFα standard (80, 40, 20, 10, 5, and 2.5 pg/mL). For each well, 50 μL of standards or samples were added (blank wells contained assay buffer only), followed by 100 μL of HRP-conjugated detection antibody. Plates were sealed and incubated at 37 °C for 60 min. Wells were washed five times with wash buffer (350 μL per wash). Substrate A (50 μL) and Substrate B (50 μL) were then added and incubated for 15 min at 37 °C in the dark. The reaction was stopped with 50 μL stop solution, and absorbance was read at 450 nm within 15 min using a microplate reader. TNFα concentrations were calculated from the standard curve.

### 2.7 Western blot analysis

RSC96 cells were treated under NG or H) conditions with ATV and/or the TNF signaling inhibitor R-7050 (TargetMol, Cat. No. T4637) as indicated. At the end of treatment, cells were washed twice with ice-cold PBS and lysed in RIPA buffer supplemented with protease and phosphatase inhibitors. Lysates were kept on ice for 20 min with occasional mixing and then clarified by centrifugation at 12,000 × g for 10 min at 4 °C. Protein concentrations were determined using a BCA assay, and equal amounts of protein were denatured in SDS loading buffer and separated by SDS–PAGE. Proteins were transferred onto PVDF membranes, which were then blocked in 5% BSA for phospho-proteins in TBST.

Membranes were incubated overnight at 4 °C with primary antibodies against phospho-NF-κB p65 (Ser536), total NF-κB p65, and β-actin, followed by incubation with HRP-conjugated secondary antibodies at room temperature. Immunoreactive bands were visualized using an enhanced chemiluminescence (ECL) substrate and imaged with a chemiluminescence detection system. Band intensities were quantified by densitometry using ImageJ. Phosphorylated p65 was normalized to total p65, and β-actin served as the loading control.

## 3 Results

### 3.1 Network toxicology and molecular docking

**FIGURE 2:**
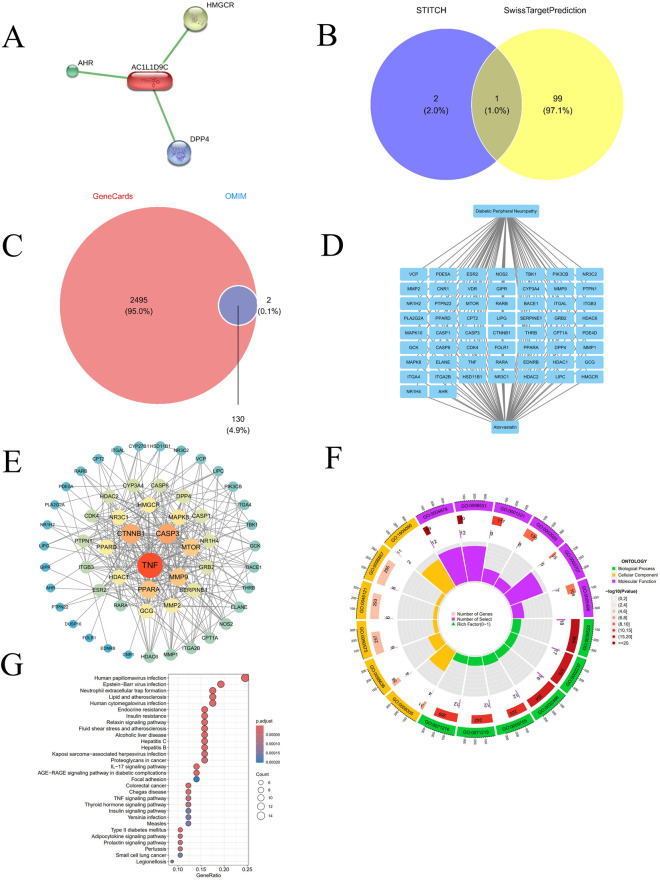
**(A)** atorvatatin target retrieved from the STITCH. **(B)** Number of atorvatatin target genes predicted by the STITCH and SwissTargetPrediction databases. **(C)** Number of DPN-related genes retrieved from the OMIM and GeneCards databases. **(D)** DPN-target gene-atorvatatin regulatory network: the upper panel represents DPN, the central panel represents target genes, and the lower panel represents atorvatatin. **(E)** Global molecular interaction network. **(F)** Circular visualization of Gene Ontology (GO) enrichment analysis for genes associated with atorvastatin and DPN, including biological processes (BP), cellular components (CC), and molecular functions (MF). **(G)** Kyoto Encyclopedia of Genes and Genomes (KEGG) pathway enrichment analysis of genes/molecules associated with atorvastatin and DPN.

Target prediction analysis showed that compound AC1L1D9C interacts with AHR, HMGCR, and DPP4, suggesting it may exert biological effects by regulating these targets and laying a foundation for further elucidating its mechanism (Figure 2A). Venn diagram analysis of target screening results from STITCH and SwissTargetPrediction databases showed that STITCH contains two unique targets (2.0%), SwissTargetPrediction contains 99 unique targets (97.1%), and there is 1 common target (1.0%) between them (Figure 2B). GeneCards and OMIM database gene screening results via Venn diagram showed that GeneCards covered 2,495 genes (95.0%), OMIM had two unique genes (0.1%), and there were 130 common genes (4.9%) between them (Figure 2C). This result suggests that atorvastatin may participate in the pathogenesis of DPN through the synergistic effects of multiple targets, providing a foundation for subsequent mechanistic studies.

The molecular association network confirmed TNF as an overlapping gene between DPN and atorvastatin (Figure 2D). The PPI network contained 57 nodes and 299 interaction edges. Topological analysis showed an average node degree of 10.491. Using a node degree ≥20 as the cutoff, 30 core targets were identified, with TNF as the core node possessing 41 edges (Figure 2E).

GO analysis yielded 1,170 terms (Figure 2F). Among these, 1,063 belonged to Biological Process, including synaptic vesicle exocytosis (GO:00071216) and neurotransmitter release, processes related to abnormal nerve conduction in DPN, and lipid metabolic processes associated with disrupted myelin homeostasis. There were 23 Cellular Component terms, such as vesicular lumen (GO:0012505) and mitochondrial membrane; dysfunction in these components may lead to impaired neurotransmitter release and mitochondrial dysfunction, related to DPN-induced hypoesthesia. Eighty-four terms were Molecular Functions, covering G protein-coupled receptor activity, linked to pain signal modulation, and lipid binding, associated with myelin impairment.

KEGG enrichment results (Figure 2G) indicated pathways grouped into metabolic disorders, inflammatory responses, and immune regulation, consistent with DPN pathology. The TNF signaling pathway was enriched (GeneRatio ∼0.10). With a significant p.adjust value and gene count, this underscores the role of TNF-mediated signaling in the underlying processes.

### 3.2 Single-cell sequencing

**FIGURE 3:**
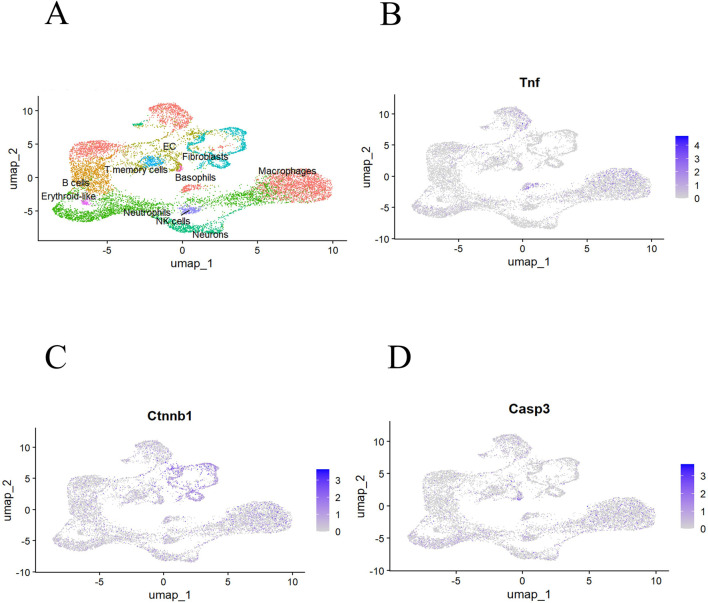
Single-cell RNA-seq analysis of candidate gene expression in an external mouse T2DM bone tissue dataset (GSE272612). **(A)** UMAP visualization showing major cell-type annotations based on canonical marker genes. **(B–D)** Feature plots showing the expression distributions of TNF, CTNNB1, and CASP3 across annotated cell populations within this dataset.

Results of single-cell RNA sequencing. To provide cell-type–associated context for candidate genes, we analyzed single-cell RNA-seq data from a mouse T2DM bone tissue dataset (GSE272612). After quality control and clustering, cells were divided into 18 clusters and annotated into 10 major cell types based on canonical marker genes, including Macrophages, Neutrophils, Fibroblasts, B cells, Endothelial Cells (EC), a neuronal cluster (neuronal-like), T memory cells, NK cells, Erythroid-like cells, and Basophils (Figure 3A). The core genes TNF, CTNNB1, and CASP3 showed cluster-dependent expression patterns, including expression in the neuronal cluster annotated based on marker genes within this dataset (Figures 3B–D). These results are presented as exploratory and reflect expression patterns within the analyzed dataset rather than definitive peripheral-nerve localization.

### 3.3 Molecular docking

**FIGURE 4:**
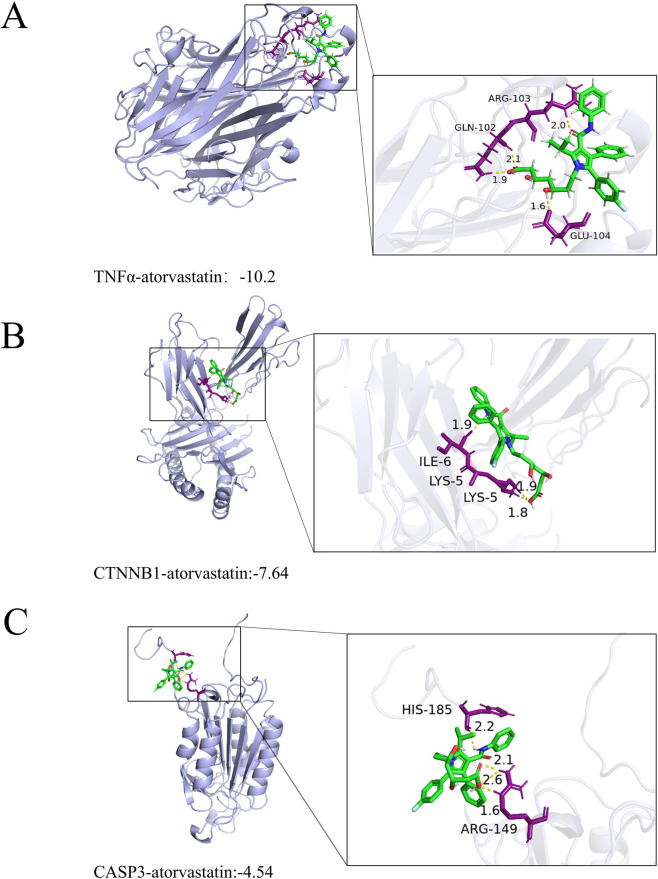
**(A)** Molecular docking and binding mode of TNFα with atorvastatin. **(B)** Molecular docking and binding mode of CTNNB1 with atorvastatin. **(C)** Molecular docking and binding mode of CASP3 with atorvastatin.

The binding energy of TNFα–atorvastatin was −10.2 kcal/mol, suggesting a relatively favorable predicted interaction. The docking pose indicated multiple potential non-covalent contacts (e.g., hydrogen bonds/salt-bridge-like interactions) involving ARG-103, GLN-102 and GLU-104 (Figure 4A). The binding energy of CTNNB1-atorvastatin was −7.64 kcal/mol, indicating a strong interaction (Figure 4B). Atorvastatin formed a stable complex with CTNNB1 via strong intermolecular interactions (e.g., hydrogen bonds and salt bridges), which involved LYS-5 (1.8 Å) and ILE-6 (1.9 Å) of CTNNB1. The binding energy of CASP3-atorvastatin was −4.54 kcal/mol, indicating a weak-to-moderate interaction (Figure 4C). Atorvastatin bound to CASP3 through strong interactions such as hydrogen bonds and salt bridges, involving HIS-185 (2.2 Å) and ARG-149 (2.5 Å) of CASP3.

### 3.4 Molecular dynamics simulation

**FIGURE 5:**
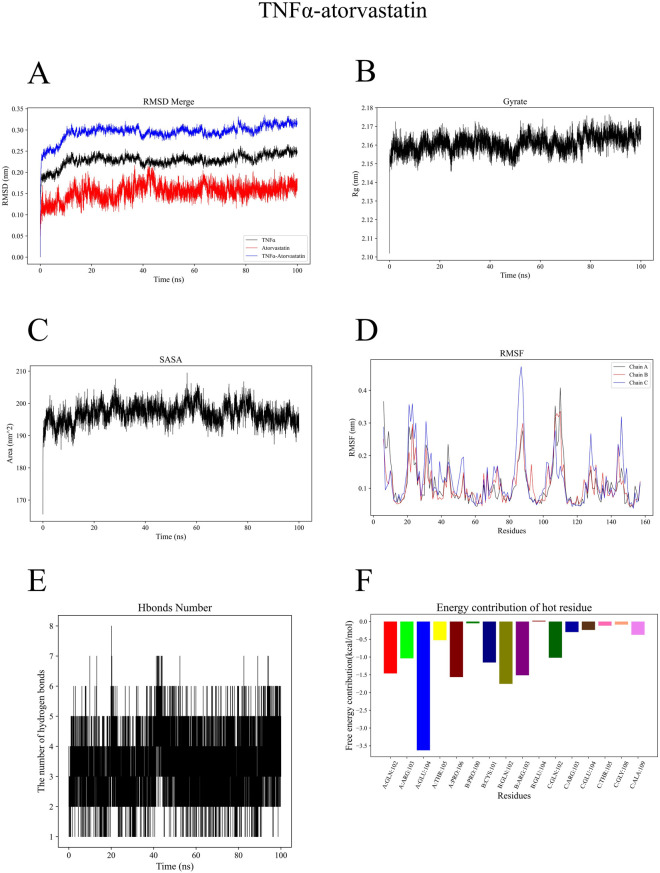
**(A)** Root Mean Square Deviation (RMSD) curve of the TNFα-atorvastatin complex during molecular dynamics simulation. **(B)** Radius of gyration curve of the TNFα-atorvastatin complex during molecular dynamics simulation. **(C)** Solvent Accessible Surface Area (SASA) curve of the TNFα-atorvastatin complex during molecular dynamics simulation. **(D)** Root Mean Square Fluctuation (RMSF) curve of the TNFα-atorvastatin complex during molecular dynamics simulation. **(E)** Hydrogen bond number variation of the TNFα-atorvastatin complex during molecular dynamics simulation. **(F)** Free energy contribution of key residues in the TNFα-atorvastatin complex.

To evaluate the contribution of each component in the experimental system, we quantified the relevant parameters and summarized the results (Table 1). TNFα-atorvastatin complex exhibited favorable structural stability, with the mean RMSD of the complex was 0.29 ± 0.02 nm, and the ligand RMSD was 0.15 ± 0.02 nm, showing that the ligand could exist stably in the binding pocket (Figure 5A). The gyration radius of the protein being 2.16 ± 0.00 nm, indicating a compact overall conformation of the system (Figure 5B). The Solvent Accessible Surface Area (SASA) value was 196.82 ± 2.97 nm^2^, indicating a moderate level of surface exposure after binding (Figure 5C). The mean RMSF of the protein was 0.12 ± 0.07 nm, suggesting low overall flexibility of residues and good structural stability (Figure 5D). Hydrogen bond analysis revealed that the complex formed an average of four protein-ligand hydrogen bonds, which provided electrostatic stabilization and spatial positioning for ligand binding (Figure 5E). In the energy decomposition results, electrostatic interaction (ΔEelec = −44.79 ± 0.45 kcal/mol) played a dominant role, followed by van der Waals interaction (ΔVDWAALS = −44.32 ± 1.29 kcal/mol), and the total free energy ΔTotal was −37.50 ± 1.35 kcal/mol, demonstrating strong binding affinity (Figure 5F).

**TABLE 1:** The contribution components of binding free energy for atorvastatin with CASP3, CTNNB1 and TNFα.

Contribution components	CASP3-atorvastatin	CTNNB1-atorvastatin	TNFα-atorvastatin
Δ_VDWAALS_	−40.84 ± 1.89	−47.98 ± 1.17	−44.32 ± 1.29
ΔE_elec_	−38.44 ± 1.13	−29.81 ± 1.50	−44.79 ± 0.45
ΔE_GB_	52.04 ± 0.85	47.50 ± 0.55	57.48 ± 0.20
ΔE_surf_	−5.78 ± 0.07	−6.84 ± 0.01	−5.87 ± 0.16
ΔG_gas_	−79.28 ± 1.20	−77.79 ± 1.90	−89.11 ± 1.33
ΔG_solvation_	46.26 ± 0.86	40.65 ± 0.55	51.61 ± 0.26
ΔTotal	−33.03 ± 1.36	−37.14 ± 1.98	−37.50 ± 1.35

**FIGURE 6:**
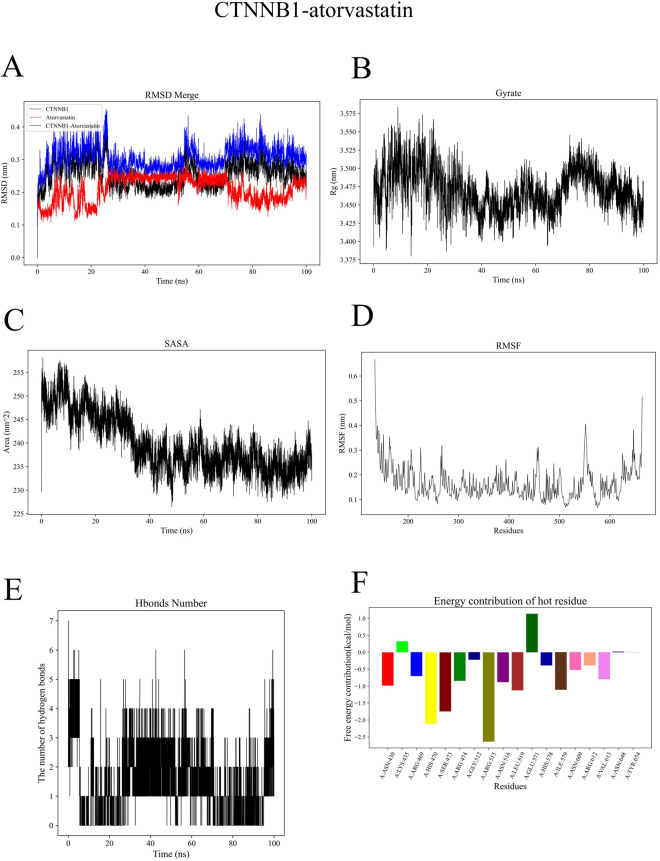
**(A)** Root Mean Square Deviation (RMSD) curve of the CTNNB1-atorvastatin complex during molecular dynamics simulation. **(B)** Radius of gyration curve of the CTNNB1-atorvastatin complex during molecular dynamics simulation. **(C)** SASA curve of the CTNNB1-atorvastatin complex during molecular dynamics simulation. **(D)** Root Mean Square Fluctuation (RMSF) curve of the CTNNB1-atorvastatin complex during molecular dynamics simulation. **(E)** Hydrogen bond number variation of the CTNNB1-atorvastatin complex during molecular dynamics simulation. **(F)** Free energy contribution of key residues in the CTNNB1-atorvastatin complex.

Simulation results confirmed the overall stability of the CTNNB1-atorvastatin complex system. The mean RMSD of the complex was 0.31 ± 0.04 nm, with the ligand RMSD being 0.21 ± 0.04 nm, indicating stable binding site structure without dissociation (Figure 6A). The gyration radius of the protein was 3.47 ± 0.03 nm, maintaining a stable level throughout the simulation (Figure 6B). The SASA value was 239.86 ± 5.93 nm^2^, indicating relatively exposed binding sites of the complex (Figure 6C). The mean protein RMSF was 0.17 ± 0.07 nm, reflecting that most residues maintained low flexibility (Figure 6D). The system formed an average of 1 hydrogen bond, suggesting that its binding stability relies more on hydrophobic interactions and van der Waals forces (Figure 6E). Results of energy decomposition showed that van der Waals interactions (ΔVDWAALS = −47.98 ± 1.17 kJ/mol) were the main driving force for ligand binding, followed by electrostatic interactions (ΔEelec = −29.81 ± 1.50 kJ/mol). The total free energy (ΔTotal) was −37.14 ± 1.98 kJ/mol, demonstrating good binding affinity of the system (Figure 6F).

The CASP3-atorvastatin complex exhibited high structural stability during the simulation. The mean Root Mean Square Deviation (RMSD) of the complex was 0.27 ± 0.02 nm, among which the RMSD of the ligand atorvastatin was low (0.11 ± 0.02 nm), suggesting that the ligand remained in a stable state without positional drift (Supplementary Figure S1A). The overall gyration radius of the protein was 1.79 ± 0.01 nm with small fluctuations, indicating good overall conformational compactness of the protein molecule (Supplementary Figure S1B). The SASA was 122.85 ± 3.20 nm^2^ (Supplementary Figure S1C). The mean Root Mean Square Fluctuation (RMSF) of the protein was 0.12 ± 0.09 nm, reflecting low overall flexibility of the residues and high stability in the binding site region (Supplementary Figure S1D). Hydrogen bond analysis showed that the complex formed an average of four protein-ligand hydrogen bonds, which provided electrostatic stabilization and spatial positioning for ligand binding (Supplementary Figure S1E). Results of energy decomposition revealed that both hydrophobic interactions (ΔVDWAALS = −40.84 ± 1.89 kJ/mol) and electrostatic interactions (ΔEelec = −38.44 ± 1.13 kJ/mol) made significant contributions to the binding energy. The final total free energy (ΔTotal) was −33.03 ± 1.36 kJ/mol, indicating a spontaneous and stable thermodynamic process (Supplementary Figure S1F).

### 3.5 Cell viability assay

**FIGURE 7:**
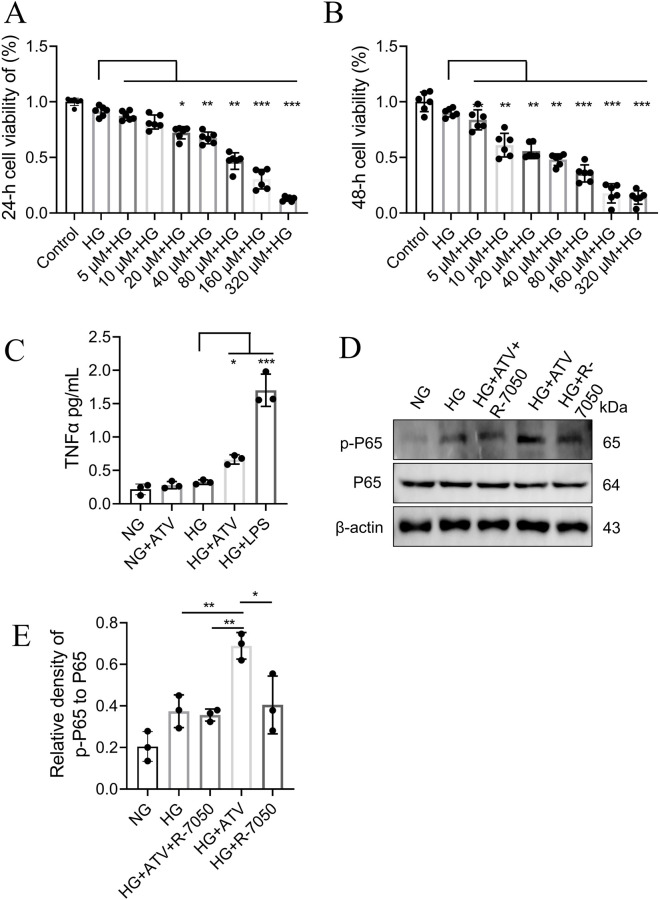
Atorvastatin enhances TNFα release and NF-κB activation in high glucose–treated RSC96 cells. **(A,B)** Cell viability measured by CCK-8 after 24 h **(A)** or 48 h **(B)** exposure to high glucose (HG) with increasing concentrations of atorvastatin (ATV; 5–320 μM). **(C)** TNFα levels in culture supernatants measured by ELISA in normal glucose (NG) or HG conditions with/without ATV; LPS was used as a positive control. **(D)** Representative immunoblots showing p-NF-κB p65 (Ser536), total p65, and β-actin. **(E)** Densitometric quantification of the p-p65 (Ser536)/p65 ratio in NG, HG, and HG-treated cells with ATV and/or the TNF signaling inhibitor R-7050. Data are presented as mean ± SD; each dot represents an independent replicate. Statistical significance is indicated in the panels (*p < 0.05, **p < 0.01, ***p < 0.001; ns, not significant).

To investigate the potential neurotoxic effect of atorvastatin in the context of DPN, we evaluated its impact on RSC 96 cell viability under high glucose conditions, which mimic the diabetic milieu. As shown in (Figures 7A,B), atorvastatin significantly inhibited cell viability in a time- and dose-dependent manner. Compared to the control group, a statistically significant reduction in cell viability was observed at concentrations as low as 20 μM after 24 h, an effect that was markedly enhanced at 48 h. Cell viability declined precipitously with increasing atorvastatin concentrations, particularly above 40 μM.

### 3.6 Enzyme-linked immunosorbent assay

At 48 h, HG (30 mM) was associated with increased TNFα secretion compared with NG (5.5 mM). Under NG conditions, atorvastatin (40 μM) showed limited/no marked effect on TNFα, whereas under HG conditions, atorvastatin co-treatment was associated with a further increase in TNFα (Figure 7C). Overall, these data suggest that atorvastatin exposure is associated with increased TNFα levels under high-glucose conditions, which may be relevant to inflammatory responses in DPN.

### 3.7 Western blot

To further examine whether inflammatory signaling was engaged under hyperglycemic conditions, we assessed NF-κB activation by measuring p65 phosphorylation (Ser536). As shown in Figure 7D, high glucose alone increased p-p65 compared with NG, accompanied by a higher p-p65/p65 ratio (Figure 7E). Under HG conditions, atorvastatin treatment led to a further rise in p-p65, resulting in the highest p-p65/p65 ratio among the tested groups. Notably, co-treatment with the TNF signaling inhibitor R-7050 attenuated the HG + ATV–associated increase in p65 phosphorylation, bringing the p-p65/p65 ratio closer to the HG level. R-7050 alone under HG conditions did not markedly elevate p65 phosphorylation compared with HG. Together, these results are consistent with the involvement of TNF/NF-κB–related inflammatory signaling in the cellular response to atorvastatin under hyperglycemic stress.

## 4 Discussion

DPN causes nerve pain and disrupts patients’ daily life. Its characteristic pathological changes include axonal loss and regeneration, myelin abnormalities and demyelination (Jack and Wright, 2012). Without early treatment, it can lead to foot ulcers, then gangrene, and even amputation (Yu, 2021). Dyslipidemia is a major risk factor for DPN in type 2 diabetes (Girach et al., 2006). Studies show that statins do more than lower lipids, they can also improve blood vessel function and reduce inflammation (Davis et al., 2008; Palinski, 2001; Zhou and Liao, 2010). This may help protect nerves and treat DPN. But epidemiological studies link statin use to a higher risk of neuropathy (Corrao et al., 2004). This study integrates network toxicology, single-cell RNA sequencing, molecular docking, and molecular dynamics simulations to systematically identify potential targets of atorvastatin-induced DPN, such as TNF, CTNNB1, and CASP3. Studies show TNF showed high network centrality and relatively favorable predicted interaction with atorvastatin in the docking/MD analyses. Based on these observations together with the *in vitro* readouts, our results are consistent with a potential involvement of TNF/TNFα-associated responses in atorvastatin-related cellular stress under the tested conditions.

In this study, GO and KEGG enrichment analyses identified TNF, CTNNB1, and CASP3 as key genes associated with the mechanism of atorvastatin-induced DPN. These targets were enriched in critical biological processes such as oxidative stress, neurotransmitter release, and lipid metabolism. Previous studies have indicated that lipophilic statins can induce mitochondrial damage through multiple mechanisms. This triggers oxidative stress, resulting in cellular injury (Kaufmann et al., 2006). Our findings are consistent with this pathway. KEGG analysis indicates that atorvastatin may promote DPN through the AGE-RAGE signaling pathway. Pathway activation recruits mDia1 and, through the PI3K/Akt axis, promotes NF-κB nuclear translocation to regulate downstream transcription. RAGE binding to Aβ peptides on the neuronal surface triggers NF-κB activation, inducing the release of macrophage colony-stimulating factor (M-CSF) and other pro-inflammatory cytokines. M-CSF further interacts with RAGE, amplifying oxidative stress and inflammatory responses (Bhattacharya et al., 2023; Derk et al., 2018). This pathway represents a potential mechanism for atorvastatin-induced DPN. We have centered our discussion on the connections between atorvastatin, its targets, and DPN, focusing on inflammation, oxidative stress, and metabolic disturbances to hypothesize potential underlying pathways. Molecular docking suggested that atorvastatin may form a plausible binding pose with TNFα, CTNNB1, and CASP3, and the predicted binding scores for TNFα were comparatively favorable. These computational results provide supportive, hypothesis-generating evidence for a possible interaction, but additional experimental work is required to determine whether such interactions translate into inflammatory/oxidative-stress changes and peripheral nerve injury *in vivo*. Notably, prior structural and biochemical studies have shown that small molecules can directly engage soluble TNFα and modulate its trimer stability, for example by accelerating subunit dissociation and thereby reducing receptor-competent TNFα. These reports support the general plausibility that low-molecular-weight ligands may interact with TNFα. Nevertheless, whether atorvastatin binds TNFα under physiological conditions and whether such binding leads to functional modulation remain to be determined, and our docking/MD results should be interpreted as hypothesis-generating (He et al., 2005).

TNF, a key inflammatory mediator, is associated with the pathological progression of neuropathy in type 2 diabetes (Hussain et al., 2013). Among its subtypes, TNFα serves as the central functional molecule within the TNF family and plays significant roles in neuroinflammation, apoptosis, and tumorigenesis (Zelová and Hošek, 2013). Meta-analyses of database studies have revealed that serum levels of TNFα are significantly elevated in patients with DPN compared to healthy controls and type 2 diabetic patients without DPN (Mu et al., 2017). Under hyperglycemic conditions, upregulated expression of inflammatory mediators including TNFα and NF-κB not only impairs mitochondrial function and neurotrophic support, but also damages microvascular integrity via protein kinase C activation and enhanced hexosamine pathway activity, accelerating the progression of peripheral neuropathy (Mendez et al., 1996; Morohoshi et al., 1996; Vlassara et al., 1988; Xue et al., 2021). Mechanistic studies demonstrate that modulating the TNFα pathway can ameliorate DPN. Xiaoke Bitong Capsule (XBC) has been shown to alleviate neuroinflammatory damage in model animals by suppressing TNF signaling (Tian et al., 2024). Photobiomodulation (PBM) therapy reduces pain and nerve injury by decreasing TNFα expression in the central nervous system (Ferreira et al., 2024). Mudan Granules used in clinical management of DPN, reported/predicted to interact with TNFα (Long et al., 2025). These findings support the relevance of TNFα in DPN treatment. The relationship between statins and DPN involves complex mechanisms. Studies indicate that atorvastatin at higher concentrations, may induce neurotoxicity through multiple pathways: they promote macrophage infiltration into adipose tissue, increasing TNFα production, and impair mitochondrial function by disrupting calcium homeostasis, inhibiting the electron transport chain, and inducing mitochondrial membrane potential collapse (Golbidi and Laher, 2014; Murinson et al., 2012; Sirvent et al., 2005). These alterations lead to reduced ROS clearance, increased electron leakage, and subsequent oxidative stress (Esposito et al., 1999; Kaufmann et al., 2006). Oxidative stress and TNFα expression form a vicious cycle, wherein ROS promotes TNFα generation via activation of pathways such as NF-κB, while elevated TNFα further exacerbates oxidative stress (Husain et al., 2015; Ozsoy et al., 2008). Additionally, statin-induced depletion of coenzyme Q10 not only compromises mitochondrial antioxidant capacity but also elevates circulating levels of inflammatory factors including TNFα, collectively contributing to peripheral nerve damage (Banach et al., 2015; Duberley et al., 2014; Hou et al., 2023). These mechanisms together constitute the molecular basis by which statins may induce or exacerbate DPN.

Neuropathic pain is a key clinical feature of DPN, and sensory neuron–specific mechanisms are increasingly recognized. In line with the increasing focus on sensory neuron–related mechanisms, transcriptomic profiling of human dorsal root ganglia (DRGs) from patients with painful DPN has reported an inflammatory gene signature (including macrophage-associated transcripts) together with reduced expression of multiple neuronal genes, suggesting that neuroimmune interactions within the ganglion may contribute to pain hypersensitivity. These human data provide a clinical context in which TNF/NF-κB–related inflammatory signaling may be relevant, although they do not establish a direct causal link with atorvastatin exposure. VGLUT2-positive sensory neurons have been proposed as major carriers of peripheral nociceptive signaling to the spinal cord, and VGLUT2-associated changes have been reported in STZ-induced DPN models. Moreover, aberrant activation of the sTNF/TNFR1 axis in VGLUT2+ neurons has been implicated in neuroinflammatory regulation through NF-κB signaling. In this study, we provide *in vitro* support for involvement of the TNF/NF-κB pathway by showing increased phosphorylation of NF-κB p65 under HG + ATV conditions and its attenuation by R-7050. However, VGLUT2 expression changes and TNF–VGLUT2 relationships were not directly assessed here and warrant targeted validation in sensory neuron models and/or *in vivo* DPN settings (Zhang et al., 2024). Oxidative stress and inflammation are tightly linked in DPN pathogenesis. PRDX6 has been proposed as a redox-regulatory molecule with both antioxidant and anti-inflammatory properties and may represent a candidate node connecting ROS homeostasis with TNF/NF-κB signaling. In the present study, we provided experimental support for activation of the TNF/NF-κB pathway by showing increased p65 phosphorylation under HG + ATV conditions and its attenuation by R-7050. However, PRDX6 expression/activity (GPx/iPLA2) and its potential relationship with TNF/NF-κB activation were not examined and warrant targeted investigation in future work (Cao et al., 2022).

CTNNB1 encodes a multifunctional protein that is critically involved in cellular adhesion, signal transduction, and proliferation. It is highly expressed in brain regions such as the cerebral cortex, hippocampus, and cerebellum, playing a pivotal role in early brain development and has been strongly implicated in neurodevelopmental disorders, including intellectual disability, schizophrenia, and autism spectrum disorder (Zhuang et al., 2023).Pathogenic variants in the CTNNB1 gene are also associated with the pathogenesis of primary aldosteronism(PA) (Teo et al., 2015). In PA, adrenal overproduction of aldosterone leads to dyslipidemia, a key risk factor for DPN, suggesting a potential mechanism whereby CTNNB1 may contribute to DPN through the modulation of lipid metabolism (Seidel et al., 2019). At the molecular level, CTNNB1 acts as a central effector and transcriptional co-activator within the canonical Wnt signaling pathway (Liu et al., 2022). Within the hyperglycemic milieu, the Wnt pathway drives DPN progression through its regulation of Schwann cell apoptosis; pathway activation promotes cell immortalization, and its inhibition conversely compromises cell viability (Liu et al., 2020). GSK3β serves as a critical node, bridging the Wnt and PI3K/AKT signaling pathways to indirectly regulate Schwann cell apoptosis and thereby influence DPN pathogenesis (Liu et al., 2016). Based on these findings, we hypothesize that atorvastatin may contribute to DPN development by targeting CTNNB1. Furthermore, crosstalk exists between TNFα and β-catenin signaling; TNFα activates the β-catenin pathway via a signaling cascade initiated through the TNFR1 death domain, thereby promoting adipogenesis (Cawthorn et al., 2007; Kramer et al., 2023). This suggests a plausible mechanism through which TNFα may also accelerate the progression of DPN.

CASP3 serves as a pivotal executioner protease in the apoptotic pathway. In diabetic kidney disease (DKD) models, elevated CASP3 levels are closely associated with enhanced oxidative stress and inflammatory apoptosis (Yu et al., 2025). This enzyme executes its function through proteolytic cleavage of key substrates including poly(ADP-ribose) polymerase (PARP) (Salvesen and Dixit, 1997). Pathologically, CASP3 contributes to neurodegenerative processes, where its pharmacological inhibition effectively reverses HIV-1-induced TDP-43 aggregation and associated neurotoxicity (Yang et al., 2024). In DPN, Schwann cell apoptosis represents a central pathological event, and inhibition of activated CASP3 can partially ameliorate both autophagy suppression and apoptotic death in these cells (Zhang et al., 2021). Furthermore, *in vitro* studies confirm that atorvastatin triggers mitochondrial cytochrome c release, subsequently activating CASP3, which then cleaves essential proteins involved in RNA splicing, DNA repair, and other critical cellular processes, ultimately inducing apoptotic cell death (McIlwain et al., 2013; Panou et al., 2024). Based on current evidence, we propose a mechanistic hypothesis wherein atorvastatin promotes DPN progression via CASP3 activation: atorvastatin directly targets mitochondria, disrupting membrane integrity and inducing cytochrome c release into the cytosol. This process activates the caspase cascade, upregulating the “executioner” CASP3, which subsequently triggers apoptosis in peripheral neurons or glial cells (such as Schwann cells), thereby accelerating DPN pathogenesis.

From the perspective of neuronal excitability regulation, voltage-gated sodium channels (VGSCs) occupy a pivotal position in action potential propagation and represent crucial therapeutic targets for chronic pain management (Bennett et al., 2019). In DPN, TNFα has been demonstrated to directly activate the Nav1.7 subtype of sodium channels, thereby contributing to the development of neuropathic pain (Huang et al., 2014). Concurrently, disruption of calcium homeostasis plays an essential role in disease pathogenesis (Fernyhough and Calcutt, 2010). Downregulation of the mitochondrial calcium uniporter (MCU) compromises cellular calcium buffering, exacerbates oxidative stress, and promotes calcium influx, collectively facilitating apoptosis through both direct signaling and indirectly via induction of mitochondrial dysfunction (Jiang et al., 2025; Osmanlıoğlu and Nazıroğlu, 2024). Sustained calcium overload further activates the mitochondrial permeability transition pore (mPTP), causing collapse of the mitochondrial membrane potential and cytochrome c release, which initiates the caspase-dependent apoptotic cascade (Li et al., 1997). Taken together, our analyses suggest that atorvastatin exposure is associated with cellular stress/injury-related signals under the tested experimental conditions, and TNF emerged as a prioritized candidate target from the integrated analyses. As a selective, competitive HMG-CoA reductase inhibitor, atorvastatin is widely used to regulate hyperlipidemia. Its lipophilic nature allows broad distribution in the body and direct access to HMG-CoA reductase, making it a preferred clinical choice (Haj Hussein et al., 2022). Although the pathogenic mechanism of DPN remains unclear, extensive clinical data indicate that DPN can be triggered by mild inflammation, intraepidermal nerve fiber degeneration, impaired blood supply, hyperglycemic toxicity, and autoimmune disorders. Among these, low-grade autoinflammation caused by hyperglycemia, mitochondrial dysfunction, and dyslipidemia is a significant contributing factor. Thus, as a lipid-lowering agent, atorvastatin has the potential to mitigate the onset of DPN. However, clinical applications have revealed that some patients developed peripheral neuropathy—such as limb numbness, paresthesia, and burning pain—after taking statins (Mulchandani et al., 2018). Our team employed network toxicology and molecular docking to identify potential interaction targets between atorvastatin and DPN. Key targets were screened and their relevance demonstrated, elucidating the mechanistic basis of atorvastatin’s toxic effects on DPN.

To validate the predictions from network toxicology and molecular docking experiments, and to further elucidate the association between atorvastatin-induced neurotoxicity and the TNFα signaling pathway, we conducted cell-based assays. These experiments were performed under high-glucose conditions to simulate the diabetic microenvironment, using RSC 96 cells as a representative model for neural cells. ELISA and CCK-8 assays demonstrated that in a high-glucose environment, atorvastatin-treated RSC 96 cells exhibited significantly elevated levels of TNFα. Furthermore, under high-glucose conditions, atorvastatin exerted cytotoxic effects on RSC 96 cells in a time- and dose-dependent manner. These findings are consistent with atorvastatin-associated cytotoxicity under high-glucose conditions and suggest a possible association with TNFα elevation. However, TNFα blockade/knockdown studies are required to determine whether TNFα is necessary for the observed effects.

The findings of this study may provide useful information for future investigations into atorvastatin-related cellular responses in the context of DPN. However, several limitations should be noted. First, our network toxicology and molecular docking analyses are mainly based on predicted molecular interactions and cellular-level evidence, and therefore may not fully capture the complexity of *in vivo* pathophysiology. In addition, bioinformatics-based target identification depends on the completeness and annotation quality of public databases and on the chosen filtering criteria; thus, false positives/negatives cannot be fully excluded, and enrichment results should be interpreted as hypothesis-generating rather than definitive. Likewise, molecular docking and molecular dynamics simulations provide *in silico* estimates of plausible binding poses and interaction stability under simplified assumptions. Predicted docking scores/energies do not directly demonstrate physical binding in a biological milieu, nor do they establish functional modulation of the target. Therefore, any inferred “modulatory role” from ligand–protein interactions should be considered preliminary and requires orthogonal experimental validation (e.g., biophysical binding assays and/or target perturbation studies. In addition, no *in vivo* DPN model was included in the present study; therefore, the translational relevance of the observed associations remains to be determined and warrants validation in established diabetic neuropathy animal models (e.g., STZ-induced or db/db models) with functional and pathological endpoints. Neuron-specific validation, including assessment of VGLUT2 expression and potential TNF–VGLUT2 interactions relevant to pain signaling, was not performed in the current study and should be addressed in future work. Effects observed under controlled conditions may be amplified or attenuated in intact organisms, and clinical confounding factors such as patient heterogeneity (e.g., genetic background, duration of diabetes, glycemic control status) and concomitant medications cannot be addressed in the current design. Furthermore, we did not systematically investigate dose–response relationships or temporal dynamics. *In vitro* experiments often use relatively high drug concentrations to elicit measurable changes within a practical timeframe, which may not reflect chronic exposure conditions in patients. We also did not assess PRDX6 expression/activity (GPx/iPLA2) or integrate oxidative stress readouts (e.g., ROS) with TNF/NF-κB signaling, and therefore the proposed redox–inflammation crosstalk remains to be tested. Importantly, we did not perform TNFα blockade/knockdown experiments in this study; therefore, a causal requirement of TNFα for the observed cytotoxicity cannot be established, and future work using pharmacological inhibition or siRNA will be necessary.

## 5 Conclusion

This study combined network toxicology, scRNA-seq analysis, molecular docking/molecular dynamics simulations, and *in vitro* experiments to explore potential atorvastatin-associated cellular responses relevant to diabetic peripheral neuropathy (DPN). TNFα, CTNNB1, and CASP3 emerged as prioritized candidates from the integrated computational analyses, with enrichment in pathways related to inflammation and oxidative stress. The external scRNA-seq dataset suggested that these genes are expressed across multiple cell types, including a neuronal/neuronal-like cluster and other cells involved in tissue homeostasis. *In silico* docking/MD analyses indicated a plausible interaction between atorvastatin and these targets, with TNFα showing relatively strong predicted affinity; however, such interactions should be considered hypothesis-generating and require orthogonal validation. In high glucose–challenged RSC96 Schwann cells, atorvastatin was associated with reduced cell viability and increased TNFα-related inflammatory readouts, and our downstream assessment of NF-κB signaling (p65 phosphorylation) provided supportive evidence that TNF/NF-κB activation may be involved under the tested *in vitro* conditions.

Overall, our findings are consistent with the possibility that atorvastatin may contribute to DPN-relevant cellular stress through TNF/TNFα-associated inflammatory signaling, while additional pathways (including those related to oxidative stress and apoptosis) may also participate and should be examined in future work. These results may help motivate further mechanistic and translational studies aimed at evaluating atorvastatin safety in diabetic neuropathy settings, including *in vivo* validation with functional endpoints. Given the predominantly computational and *in vitro* design, conclusions regarding causality and clinical implications should be drawn cautiously.

## Data Availability

The datasets presented in this study can be found in online repositories. The names of the repository/repositories and accession number(s) can be found in the article.

## Supplementary material

SUPPLEMENTARY FIGURE 1
**(A)** Root Mean Square Deviation (RMSD) curve of the CASP3-atorvastatin complex during molecular dynamics simulation. **(B)** Radius of gyration curve of the CASP3-atorvastatin complex during molecular dynamics simulation. **(C)** Solvent Accessible Surface Area (SASA) curve of the CASP3-atorvastatin complex during molecular dynamics simulation. **(D)** Root Mean Square Fluctuation (RMSF) curve of the CASP3-atorvastatin complex during molecular dynamics simulation. **(E)** Hydrogen bond number variation of the CASP3-atorvastatin complex during molecular dynamics simulation. **(F)** Free energy contribution of key residues in the CASP3-atorvastatin complex.
