# Supplementary figures and images for: Inflammatory and neurotoxic risk of atorvastatin in diabetic peripheral neuropathy: TNF-centered evidence integrating network toxicology, scRNA-Seq, and cell validation
Source: Front Chem. 2026 Feb 18;14:1739085. doi: 10.3389/fchem.2026.1739085 (PMC12956307; doi:10.3389/fchem.2026.1739085)

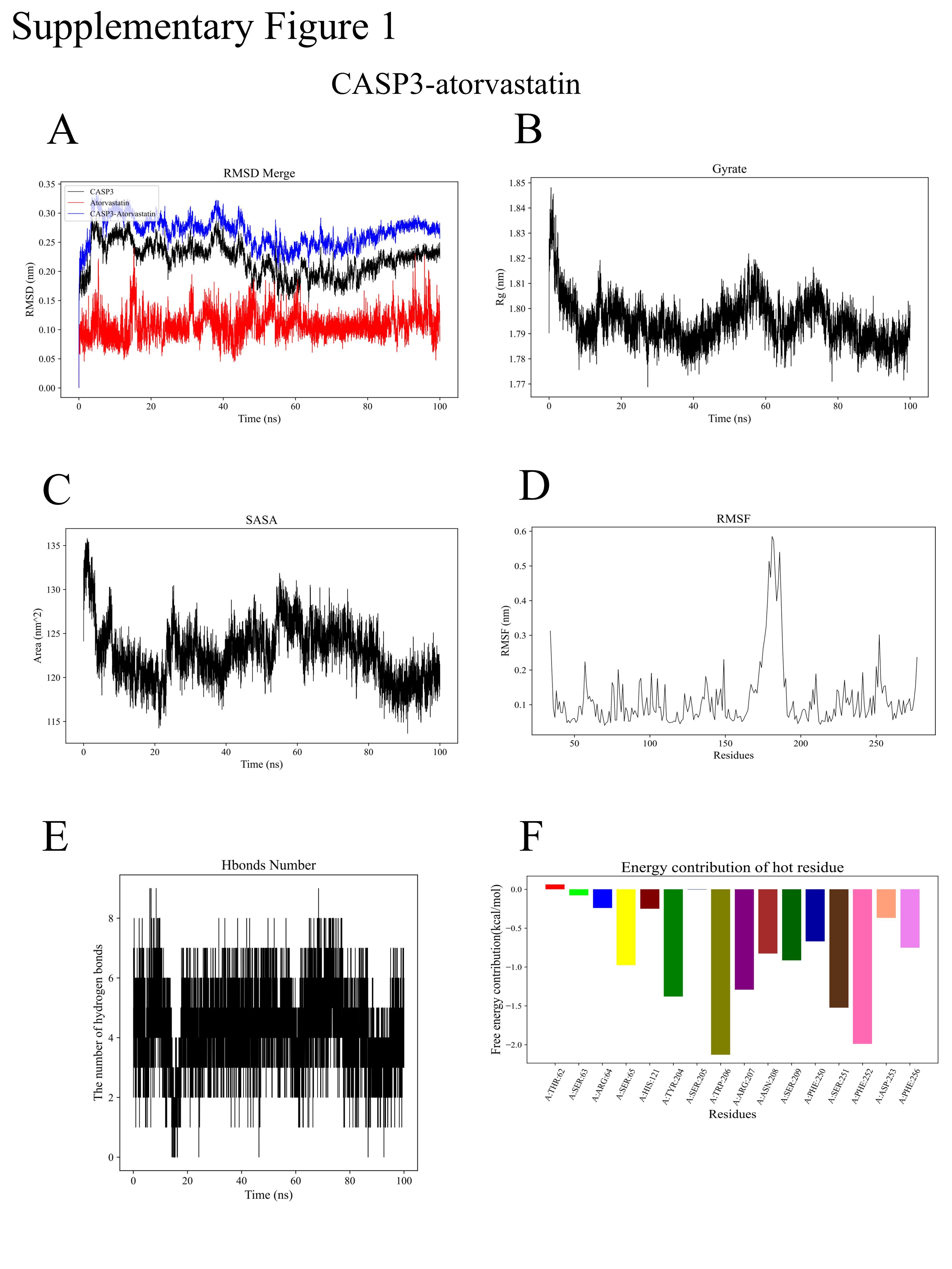

Supplement: Supplementary file 1 [file Image1.jpeg]

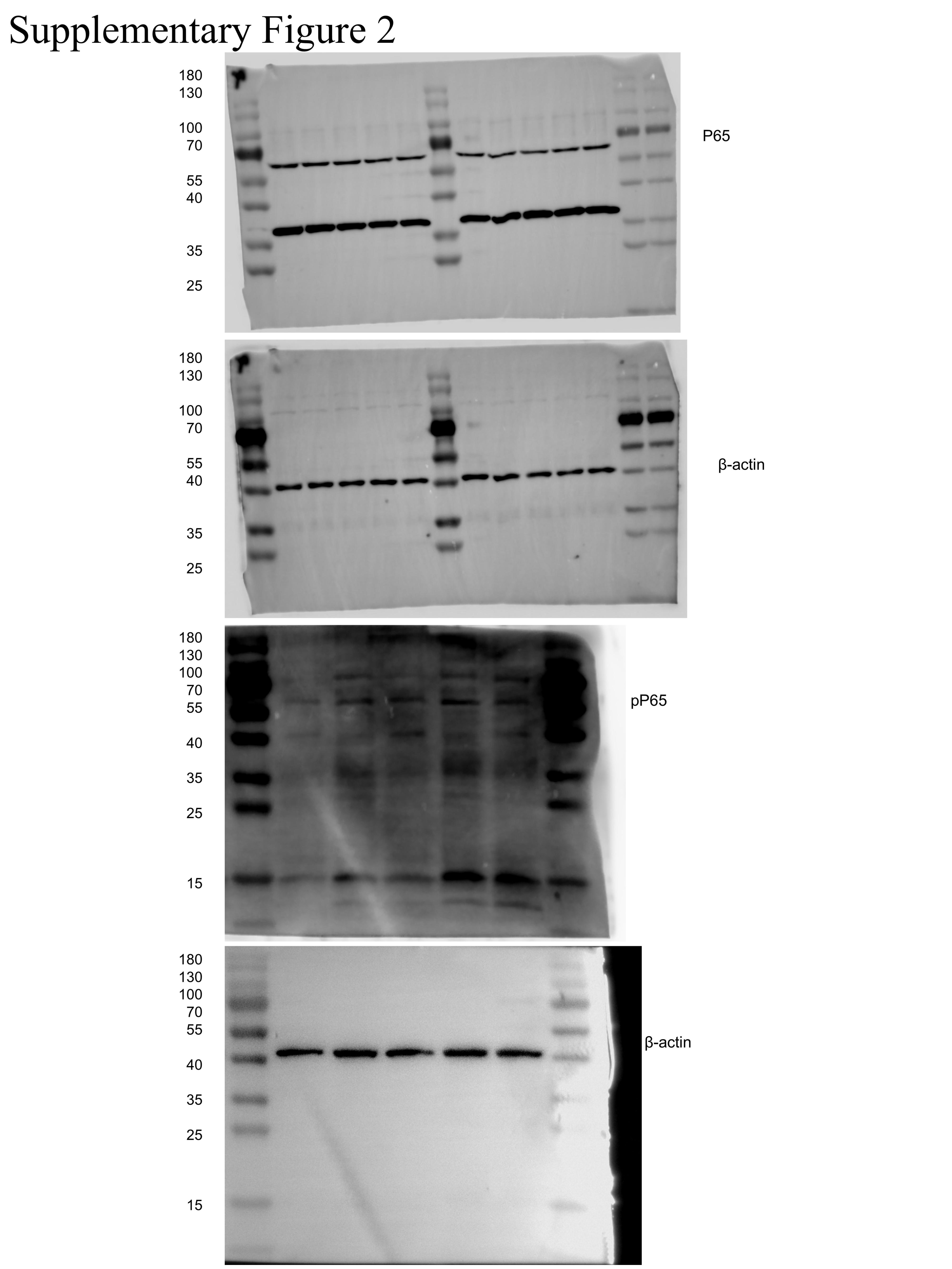

Supplement: Supplementary file 2 [file Image2.jpeg]
